# Fossil dragonfly-type larva with lateral abdominal protrusions and implications on the early evolution of Pterygota

**DOI:** 10.1016/j.isci.2021.103162

**Published:** 2021-09-24

**Authors:** Joachim T. Haug, Patrick Müller, Carolin Haug

**Affiliations:** 1 Ludwig-Maximilians-Universität München (LMU Munich), Biocenter, Großhaderner Str. 2, 82152 Planegg-Martinsried, Germany; 2 GeoBio-Center at LMU, Richard-Wagner-Str. 10, 80333 München, Germany; 3 Kreuzbergstr. 90, 66482 Zweibrücken, Germany

**Keywords:** Paleontology, Entomology, Evolutionary history, Paleobiology

## Abstract

Aquatic larvae are known in three early branches of Pterygota: Ephemeroptera (mayflies), Plecoptera (stoneflies), and Odonata (dragonflies, damselflies). A common origin of these larvae has been suggested, yet also counterarguments have been put forward, for example, the different position of larval gills: laterally on the abdomen in Ephemeroptera, terminally in Odonata, variably in Plecoptera. We discuss recent fossil findings and report a new dragonfly-type larva from Kachin amber (Myanmar), which possesses ancestral characters such as a terminal filum, maintained in ephemeropterans, but lost in modern odonatan larvae. The new larva possesses lateral protrusions on the abdominal segments where in other lineages gills occur. Together with other fossils, such as a plecopteran retaining lateral gills on the abdomen, this indicates that lateral protrusions on the abdomen might have well been an ancestral feature, removing one important argument against the idea of an aquatic larva in the ground pattern of Pterygota.

## Highlights

• A new dragonfly-type larva was found in Kachin amber (Myanmar, 99 million years old).
• The larva possesses a terminal filum, which is not known in modern dragonfly larvae
• It also exhibits lateral abdominal protrusions where in other lineages gills occur
• This find makes an aquatic larva in the ground pattern of Pterygota more likely

## Graphical abstract

**Figure:**
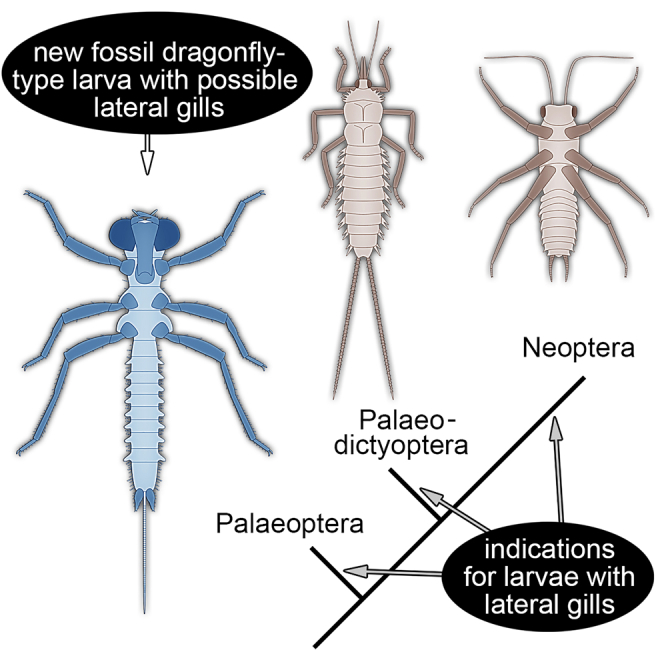

## Introduction

Our modern terrestrial ecosystems are dominated by a single group of animals, concerning species richness, individual richness, and biomass. This group is Pterygota (Grimaldi and Engel 2005, their Figures 1.3 and 1.6), “flying insects”, although quite a number of species cannot fly at all; even those that can fly, do not fly for the larger part of their life. Mostly, only the short-lived adults can fly, while immature stages lead a quite different life. The only well-known exception is Ephemeroptera (mayflies), where also the last life stage before the adult (subimago) can fly (e.g., Edmunds and Waltz 1996). However, also in mayflies the vast majority of the life is spent flightless: adult and subimago live for only few days, whereas the immature lives for several years in aquatic environments (e.g., Beutel et al., 2014), swimming or digging instead of flying.

From early on in evolution, the lifestyle strategies of immatures and corresponding adults of Pterygota were rather diverging: adults specialized on flying, immatures did not do so (e.g., Truman and Riddiford 2019). The central question about the early evolution of the group Pterygota, therefore, is how the lifestyle of the immatures looked like.

Three major extant lineages of Pterygota have immatures living in aquatic environments and are larvae from an ecological point of view, but are often addressed as naiads (see discussion in Haug 2020). These lineages are Ephemeroptera (mayflies), Odonata (damselflies and dragonflies), and Plecoptera (stoneflies). Ephemeroptera and Odonata are most likely sister groups and together form the group Palaeoptera, which is sister group to Neoptera. Within Neoptera, Polyneoptera is sister group to the other ingroups of Pterygota, and Plecoptera is an early branch within Polyneoptera (e.g., Sharma 2019; Wipfler et al., 2019).

The fact that three of the early branches of Pterygota have aquatic larvae has led to the idea that aquatic larvae may represent the ancestral state for the entire group (e.g., Kukalová-Peck 1978; 1987; Shear and Kukalová-Peck 1990; Marden and Kramer 1994; Thomas et al., 2000; Zwick 2009) that became lost in further derived ingroups (at least twice). Yet, it has also been suggested that the aquatic larvae evolved independently in these lineages (e.g., Gullan and Cranston 2010; Bitsch 2012; Garwood et al., 2012), following different lines of argumentation. For example, newer phylogenetic reconstructions indicate that Plecoptera is indeed not the sister group of the remaining lineages within Polyneoptera, but a deeper ingroup (Wipfler et al., 2019). This would either require assuming additional independent losses of aquatic larvae, or a loss in the ancestor of Neoptera and a reoccurrence in Plecoptera. In the same line of argument, the group Palaeodictyopteroidea, an extinct lineage of Pterygota only known from Palaeozoic fossils, has been suggested to have had terrestrial immatures (e.g. Grimaldi and Engel 2005; Prokop et al., 2016). Yet, newer findings indicate possible aquatic immatures also in this lineage (Prokop et al., 2019).

Another challenge to the idea of an ancestral aquatic larva for Pterygota is presented by the structure and position of the gills of the aquatic larvae (e.g., Bradley et al., 2009). In ephemeropteran larvae, gills are positioned laterally on the abdominal segments and often appear paddle-like (e.g., Edmunds and Waltz 1996). In odonatan larvae, the ancestral condition appears to have been leaf-like gills on the terminal end, although this became further derived within the group (there are some examples of lateral gills, but also of rectal respiration; see e.g. discussion in Schädel et al., 2020). In Plecoptera, the character appears to be quite variable, often tuft-like; gills may often occur on thoracic segments, but also on abdominal segments of large-sized individuals (Zwick 2009). However, the structure of these abdominal gills has been suggested to be quite different from that of ephemeropteran larvae, and such gills are also not part of the ground pattern of Plecoptera (Zwick 2009). This provides an impression of, at least, three rather different systems for gas exchange in these larvae, not further supporting a common origin. Zwick (2009) also emphasized the lack of fossil representatives to further support the presence of similar appearing gills.

We here present a new fossil larva of the lineage toward dragonflies and damselflies from Kachin amber, Myanmar (c. 99 million years old) which possesses lateral protrusions on the abdominal segments. We incorporate this and other fossils into an argumentation frame to re-evaluate whether gill structures of aquatic larvae of early representatives of Pterygota could be derived from a single origin.

## Results

**Figure 1:**
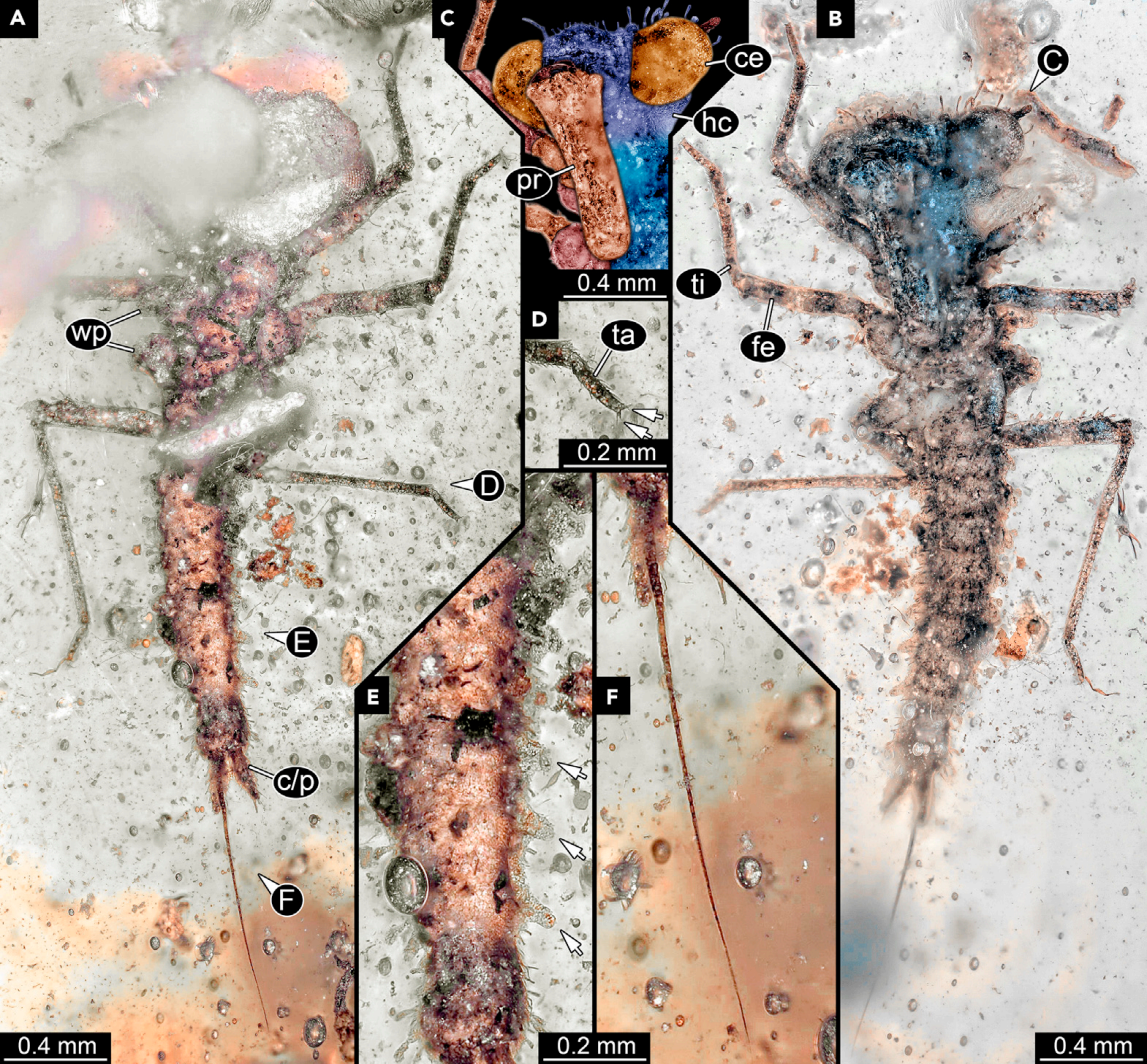
Larva of Odonatoptera, SNSB-BSPG 2021 XII 4 (originally BUB 4000) (A) Dorsal view; parts of the larva obscured by disturbances in the amber. (B) Ventral view. (C) Detailed view on the head in ventral view; color-marked and matrix virtually removed. (D) Close-up on the distal part of hind leg; arrows mark claw. (E) Close-up on the abdomen in dorsal view; arrows mark lateral protrusions. (F) Close-up on the terminal filament. Abbreviations: c/p = cercus/paraproct; ce = compound eye; fe = femur; hc = head capsule; pr = prementum (further proximal parts of labium not visible); ta = tarsus; ti = tibia; wp = wing pad.

Small larva, about 2.2 mm long (Figures 1A and 1B). Body with distinct head and trunk; trunk further differentiated into anterior part with three segments (thorax) and posterior part with eleven segments (abdomen).

Head very wide (almost 2x as wide as long), with prominent, globose compound eyes (Figure 1C). Head surface with few, prominent club-like setae (exact arrangement not discernible). Compound eyes with numerous ommatidia, in ventral view with at least 21 rows of lenses (estimated from anterior to posterior) with at least 25 lenses per row (estimated from median to lateral).

Antennae barely visible, broken off distally. Further posterior head appendages concealed by appendages of last head segment, conjoined to form prominent labium (labial mask). Visible main part (prementum; Figure 1C) as long as head is wide, distally bearing a pair of hook-like labial palps.

Thorax slightly longer than head. Each thoracic segment ventrally with a prominent pair of appendages (legs). Appendages sub-similar, subdivided into five elements. Element 1 (coxa) about as wide as long, distally tapering. Element 2 (trochanter) shorter and narrower than coxa. Element 3 (femur) 2x the length of coxa + trochanter, slightly broader than trochanter, with few setae. Element 4 (tibia) slightly longer than femur but narrower, with numerous short setae on entire surface. Element 5 (tarsus) shorter than tibia, only 30%; with few, but also short setae. Distally with a pair of claws (Figure 1D). Thoracic segments 2 and 3 (mesothroax and metathorax) dorsally with a pair of laterally protruding wingpads, about as wide as long, rounded triangular in dorsal view.

Abdomen with ten large segments, forming dorsal tergites; abdominal segment 11 only apparent by long subdivided terminal filum (terminal filament, paracercus) arising postero-dorsally from trunk end. Abdomen slightly longer than 2x the width of the head. Segments tapering toward posterior. Tergite of abdominal segment 1 wider than long; tergite of abdominal segment 9 about as wide as long; abdominal segment 10 longer than wide. Abdominal segment 2–9 with a lateral, rounded cone-shaped protrusion on each side (Figure 1E); protrusions more prominent in further posterior segments. All abdominal segments with numerous small setae and few hammer-like setae. Abdominal segment 10 ventrally with a pair of posteriorly protruding structures (cerci? paraprocts?). Terminal filum thin, slightly shorter than the combined length of abdominal segments 1–10, sub-divided into numerous elements, at least 40 (Figure 1F).

## Discussion

### Identity of the new fossil

**Figure 2:**
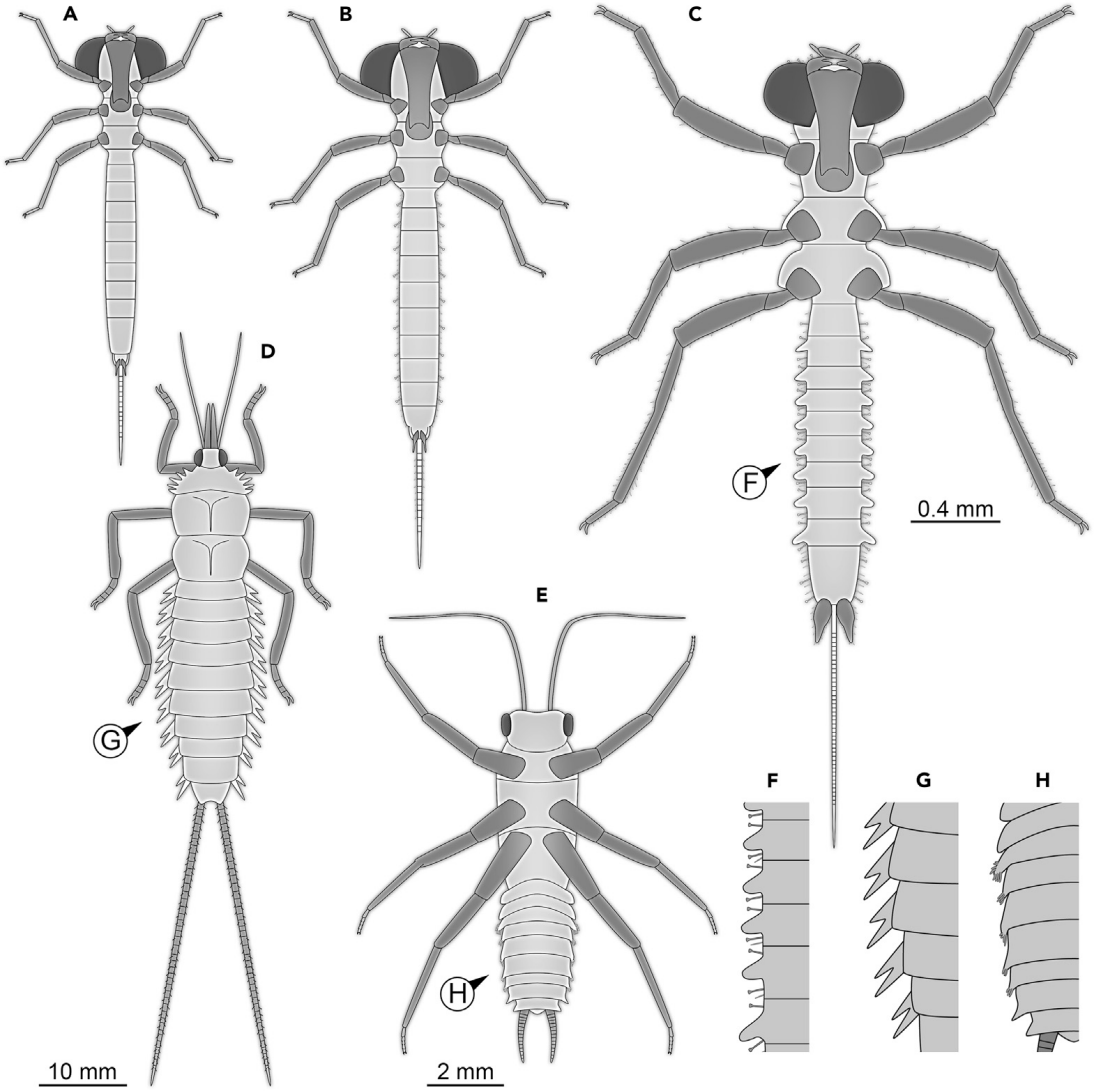
Interpretive drawings of the new larva and other fossil representatives of Palaeoptera and Plecoptera with comparable morphological structures (A and B) Different ontogenetic stages of *Arcanodraco filicauda* (based on Schädel et al., 2020). (C) SNSB-BSPG 2021 XII 4 described in this paper, presumably a later stage of *A. filicauda*. (D) Adult representative of *Corydaloides scudderi* (Palaeodictyopteroidea), wings omitted (based on Prokop et al., 2019). (E) Adult representative of *Branchioperla ianstewarti* (Plecoptera), wings omitted (based on Sroka and Staniczek 2020). (F–H) Protrusions on abdominal segments. (F) SNSB-BSPG 2021 XII 4. (G) *Corydaloides scudderi*. (H) *Branchioperla ianstewarti*.

The new fossil clearly possesses a raptorial labium, also known as labial mask. This immediately identifies it as a relative of modern dragonflies and damselflies (e.g., Bechly, 2003). Dragonfly-type larvae are still rare in Myanmar amber (Xia et al., 2015, p. 49; Zhang 2017, pp. 220, 224, and 225). Yet, one very conspicuous larva, also with a prominent labial mask, has been formally described as *Arcanodraco filicauda* (Schädel et al., 2020; Figures 2A and 2B). This larva possessed a remarkable feature, a terminal filum subdivided into numerous elements. Such a structure was only known from ephemeropteran larvae and was considered lost in the lineage of Odonatoptera (a larger group including Odonata and various additional fossil forms). Yet, the larva of *A. filicauda* demonstrates that this feature was retained in Odonatoptera and became only lost within the group (see discussion in Schädel et al., 2020). The finding emphasized that also comparably young fossils of “only” 100 million years age may well reveal such ancestral (plesiomorphic) traits.

The new fossil also clearly possesses a prominent terminal filum, but also shows numerous differences to the known specimens of *A. filicauda*. For example, the holotype of *A. filicauda* clearly does not possess the prominent lateral protrusions seen in the new fossil.

A simple comparison reveals that the new fossil is larger than the holotype of *A. filicauda*. It, therefore, seems quite possible that the new fossil represents a later developmental stage of the same species. All morphological differences between the two specimens can hence be interpreted as ontogenetic differences. This leaves no diagnostic characters to erect a new species for the new specimen. We, therefore, interpret the new specimen as a later larval stage (instar) of the species *A. filicauda* (Figure 2C). The holotype was preserved with a moulted cuticle (paratype). Based on the developed armature already this cuticle must have been at least from a stage 3 larva (see discussion in Schädel et al., 2020 for problems with the counting system of stages); the holotype is a stage 4 larva. The new specimen should therefore at least represent a stage 5 larva; based on the size differences it could even represent a stage 6 larva, as the size increase from the stage 4 larva to this specimen is relatively larger than from the presumed stage 3 to the stage 4 larva (for discussions on the relative size increase from one stage to the next, see Legaspi et al., 1994; Kutschera et al., 2012; Haug et al., 2020).

In summary, the new fossil represents a later larval stage of the earlier lineage of Odonatoptera. It retains important ancestral features (e.g., the terminal filum) that are absent in modern day representatives.

### Lateral protrusions on abdominal segments in fossils

**Figure 3:**
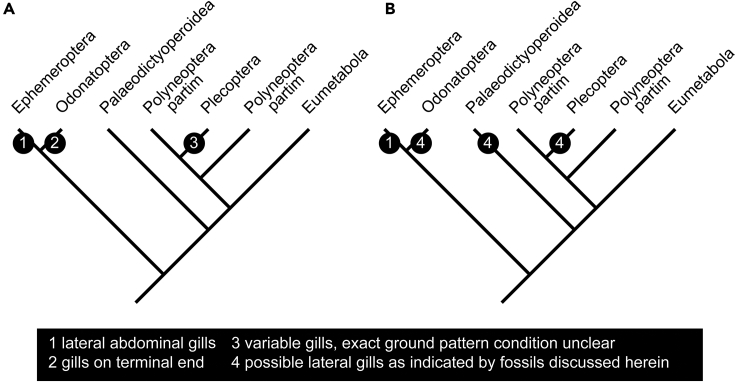
Phylogenetic tree of Pterygota (combined from Sroka et al., 2015 and Wipfler et al., 2019) with characters of gills mapped onto it (A) Condition without fossils discussed in this study. (B) Condition with fossils discussed in this study.

In fossils it is often much less easy to directly observe the function of a specific structure compared to extant organisms, for example, of presumed gill structures (e.g., Maas et al., 2009). Before further discussing the aspects of gills in early representatives of Pterygota (compare Figures 3A and 3B), we go one step back and look for lateral protrusions on abdominal segments in fossil larvae.

Many immatures of Palaedictyopteroidea possessed prominent lateral protrusions on the abdominal segments (Kukalová-Peck 1978; Garwood et al., 2012; Haug et al., 2016; Kiesmüller et al., 2019; Prokop et al. 2016, 2019). Yet, we largely lack a clear indication of these structures bearing/covering gills, or functioning in this way (Prokop et al., 2016) and in most cases, the protrusions appear to be simple lateral extensions of the tergites. Only one adult specimen preserves structures laterally on the abdomen that are reminiscent of gills (Prokop et al., 2019, their Figure 4; Figures 2D and 2G). This was interpreted as retention of larval features into the adult phase, as known in quite a number of extant species (Prokop et al., 2019, their Figure 5). Despite the uncertainty for the (few) fossil immatures of Palaeodictyopteroidea, we have at least an indirect indication of the presence of gills as lateral protrusions on the abdominal segments in such larvae (Figure 3B).

Lateral protrusions on abdominal segments are well known in mayfly larvae down to the Permian (Kukalová 1968). Generally, the similar position of the structures in the fossils and in extant larvae has led to the interpretation that already these early larvae had gills on their abdominal segments (Figures 3A and 3B; but see Prokop et al., 2016).

In a similar case to palaeodictyopteroideans, an adult fossil stonefly has been recently reported to, supposedly, retain larval gills on the anterior eight abdominal segments (Sroka and Staniczek 2020; Figures 2E and 2H). This find provided the indication of abdominal gills in fossil plecopterans (Figure 3B) that Zwick (2009) noted to be missing from the fossil record so far (Figure 3A).

For fossil odonatopteran larvae, so far the report of lateral protrusions on the abdomen was scarce, besides one specimen from the Carboniferous Mazon Creek Lagerstätte (Prokop et al., 2019). This specimen, formally named *Dragonympha srokai*, bears elongate, possibly subdivided structures postero-ventro-laterally on at least the anterior six abdominal segments (Kukalová-Peck 2009). Although the position (far ventrally) and structure (elongate, sub-divided) is quite different from the other examples, it is at least worth mentioning. The exact position of this larva within Odonatoptera remains unclear.

The new fossil specimen clearly possesses lateral protrusions on abdominal segments 2–9 (Figure 2F). This demonstrates that larvae of the early lineage of Odonatoptera, which retained obvious ancestral traits such as the terminal filament, also possessed lateral protrusions on the abdominal segments (Figure 3B). Earlier ontogenetic stages apparently lacked such structures. In other lineages, similar patterns can be observed, for example, the aquatic larvae of certain lacewings (Sisyridae) lack gills on the abdominal segments in stage 1 larvae, but possess these in stage 2 and 3 larvae.

### Integrating the new finds

The lateral protrusions on the abdominal segments in the new fossil do not bear any tufts that would support their interpretation as gills. Yet, they also do not appear pointed like the postero-lateral corners of the tergites in modern dragonfly larvae, which are spine-like for fending off predators. Instead, the protrusions appear to be separate from the tergite, as we would expect it for gills, for example in mayflies. Although these structures may not necessarily have been functional gills, they may represent the rudiments or evolutionary remnants of such gills, therefore without any tufts.

Although a lot of focus has naturally been given to older fossils, fossils from the Mesozoic can likewise provide important contributions to the reconstruction of the evolutionary history of Pterygota. This is especially true in cases where they can provide significant details not easily preserved in older types of fossil preservation, which is especially true for amber.

Fossils from Myanmar have now demonstrated the presence of lateral protrusions on abdominal segments for larvae of odonatopterans (as shown here) and indirectly also for plecopterans. This makes it again quite possible that the occurrence of lateral protrusions in immatures is an ancestral feature retained in Ephemeroptera, Plecoptera, and Odonatoptera, but lost within the latter two lineages (and other lineages of Neoptera). However, it is also possible that the lateral protrusions are ancestral for Pterygota, but became lost and reacquired independently, e.g., in Plecoptera, because of deactivation/reactivation of certain regulatory genes (e.g., Prud'homme et al., 2006; Shubin et al., 2009).

This finding cannot provide a definite answer to the question of the ancestral condition of immatures of Pterygota. Yet, it weakens, if not removes, one argument against an aquatic larva in the ground pattern of Pterygota.

### Limitations of the study

The study is based on a single specimen and few other specimens from the literature. All of these face certain phylogenetic uncertainties. This does not allow for a final conclusive statement. As always with fossils with a small sample size, the study presents now extinct morphologies and, by this, weakens certain arguments regularly used in the discussion about the early lineage of Pterygota.

## STAR★Methods

### Key resources table

**Table:** 

REAGENT or RESOURCE	SOURCE	IDENTIFIER
**Software and algorithms**		

Post-processing of all images and colour markings were performed with Adobe Photoshop CS2	Adobe Inc.	RRID:SCR_014199; URL: https://www.adobe.com/products/photoshop.html
Drawings were performed in Adobe Illustrator CS2	Adobe Inc.	RRID:SCR_010279; https://www.adobe.com/products/illustrator.html

### Resource availability

#### Lead contact

Further requests concerning the investigated material should be directed to and will be answered by the lead contact, Carolin Haug (carolin.haug@palaeo-evo-devo.info).

#### Materials availability

The specimen investigated in this study (original collection number BUB 4000) comes from the collection of one of the co-authors (PM) and was legally acquired on June 22, 2016. It is now deposited in the Staatliche Naturwissenschaftliche Sammlungen Bayerns—Bayerische Staatssammlung für Paläontologie und Geologie in Munich under repository number SNSB-BSPG 2021 XII 4. It is preserved in Cretaceous Kachin amber (c. 99 million years old) from the Hukawng Valley, Myanmar (Cruickshank and Ko 2003; Shi et al., 2012; Yu et al., 2019).

### Methods details

The specimen was documented with a Keyence VHX-6000 digital microscope equipped with a 20–2000x objective. Images were recorded under cross-polarised illumination to reduce reflections (e.g. Haug et al., 2013) and low-angle ring light and in HDR-mode (high dynamic range). Black and white background were used. Those images providing the highest contrast were used for further studies. Several images along the z-axis were recorded and fused to a fully sharp image to overcome limitations in depth of field. For high-resolution images of all details several stacked images along the x-y-axis were recorded and subsequently stitched to a panorama image. Image stacking and stitching was performed with the built-in software of the microscope. Post-processing of images and colour markings were performed with Adobe Photoshop CS2. Drawings were performed in Adobe Illustrator CS2.

## Data Availability

• All data reported in this paper will be shared by the lead contact upon request. • This paper does not report original code. • Any additional information required to reanalyze the data reported in this paper is available from the lead contact upon request.
